# Successful Use of Upadacitinib, a Selective JAK Inhibitor, in the Treatment of Two Cases of Recalcitrant Chronic Uveitis

**DOI:** 10.18502/jovr.v20.14952

**Published:** 2025-05-05

**Authors:** Diego Dominguez, Sawyer Vaclaw, Cynthia K McClard, Matlock A. Jeffries, Jila Noori

**Affiliations:** ^1^Oklahoma State University College of Osteopathic Medicine, Tulsa, OK, USA; ^2^University of Oklahoma College of Medicine, Oklahoma City, OK, USA; ^3^Cincinnati Eye Institute, Cincinnati, OH, USA; ^4^Arthritis and Clinical Immunology Program, Oklahoma Medical Research Foundation, Oklahoma City, OK, USA; ^5^Division of Rheumatology, Immunology and Allergy, Department of Medicine, University of Oklahoma Health Sciences Center, Oklahoma City, OK, USA; ^6^Veterans Affairs Medical Center, Oklahoma City, OK, USA; ^7^Department of Ophthalmology, Dean McGee Eye Institute, University of Oklahoma Health Sciences Center, Oklahoma City, OK, USA

**Keywords:** Birdshot Chorioretinopathy, JAK Inhibitors, Upadacitinib, Uveitis

## Abstract

**Purpose:**

Immunomodulatory agents, including conventional immunosuppressive treatment and biologics, are the mainstay of treating chronic uveitis. Janus kinase (JAK) inhibitors, one of the newest biologics, have shown successful outcomes in treating autoimmune diseases such as rheumatoid arthritis and inflammatory bowel diseases by suppressing the JAK/signal transducers and transcription (STAT) pathway. We present two cases of recalcitrant chronic uveitis with significant improvement in intraocular inflammation by using upadacitinib, a selective JAK1 inhibitor.

**Case Reports:**

The first case is a 59-year-old female with HLA-B27-positive Chron's disease and chronic anterior and intermediate uveitis who experienced an improvement in visual acuity, anterior chamber and vitreous inflammation, and cystoid macular edema on upadacitinib. The second patient is a 71-year-old female with birdshot chorioretinopathy, intolerant of initially used systemic immunosuppressive agents who showed significant improvement in vitreous inflammation, retinal phlebitis, and choroiditis after treatment with upadacitinib.

**Conclusion:**

Utilizing JAK inhibitors such as upadacitinib in treating uveitis, whether in isolated forms or in the context of systemic autoimmune diseases, may require further evaluation by controlled cohort studies.

##  INTRODUCTION

Noninfectious inflammatory ocular diseases can occur in isolation or in the context of systemic autoimmune diseases, such as rheumatoid arthritis (RA), juvenile idiopathic arthritis (JIA), or HLA B27-associated spondyloarthropathies. To prevent irreversible structural damage and blindness from these disorders, an appropriate therapy should be commenced promptly. Management usually starts with topical and systemic corticosteroid therapy and may lead to conventional immunomodulatory agents in cases lasting longer than three months. In cases of severe uveitis refractory to conventional immunomodulatory agents, targeted therapy with biologics such as TNF- α inhibitors and interleukin (IL) blockers has provided exciting results over the last decades. Although the current biologics in use have added a large armamentarium to treat uveitis, ongoing challenges for the management of recalcitrant uveitis urge us to explore the newer biologics in use for systemic rheumatologic diseases.

During the last decade, drugs known as JAK inhibitors or JAKinibs, blocking one or more of the molecules involved in Janus kinase (JAK)/signal transducers and activators of transcription (STAT) pathways, have been developed and tested in clinical trials for many different indications. JAK/STAT molecules are a family of cytoplasmic non-receptor protein tyrosine kinases consisting of four members, JAK1 to JAK3 and TYK2. These kinases appear to be pivotal in the transcription and expression of various critical mediators involved in immune responses, cancer development, and inflammatory diseases.^[1,2]^

Upadacitinib is a selective and reversible JAK1 inhibitor approved for treating various autoimmune diseases, such as RA, atopic dermatitis, ankylosing spondylitis, psoriatic arthritis, ulcerative colitis, and Crohn's disease.^[3,4,5,6,7,8]^ Here, we present two patients with recalcitrant uveitis with successful response to upadacitinib, a case of birdshot chorioretinopathy (BCR), and a patient with chronic anterior and intermediate uveitis associated with Crohn's disease who initiated this treatment by their rheumatologists.

**Figure 1:**
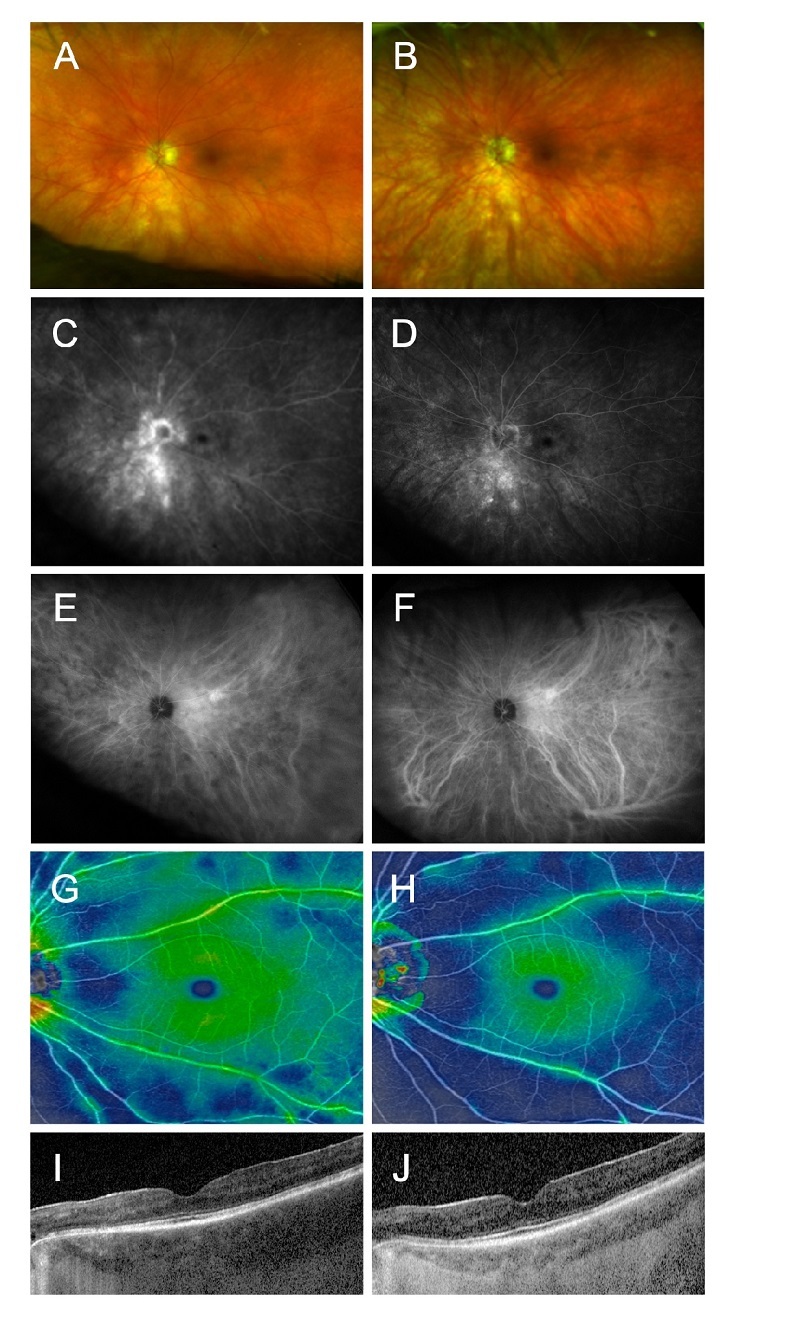
Images of the left eye before (A, C, E, G, I) and after (B, D, F, H, J) initiation of upadacitinib. Color fundus photos (A, B) and fluorescein angiography (C, D) demonstrate improvements in vitreous haze, phlebitis, and optic disc leakage. Indocyanine green angiography (E, F) demonstrates improvements in choroidal inflammation with less hypocyanescent spots. Composed color map/OCT angiography (G, H) shows improved macular and peripapillary retinal thickening, representing improved retinal vasculitis. Enhanced depth imaging optical coherence tomography of the macula (I, J) shows improvement in choroidal thickening.

##  CASE PRESENTATION

### Case 1

A 71-year-old Caucasian female was diagnosed with BCR six years ago, based on detecting yellowish subretinal lesions and positive testing for HLA A29. A thorough laboratory evaluation for systemic autoimmune diseases and infectious etiologies, including syphilis and tuberculosis, was unrevealing at that time. The patient was initially treated with an oral steroid taper and systemic immunosuppressive agents, including methotrexate at a dose of 20 mg subcutaneously weekly and, later, adalimumab 40 mg subcutaneously every other week by a rheumatologist. Both agents were discontinued due to intolerance of systemic adverse effects, including increased fatigue, flulike symptoms, and increased upper respiratory infections. She continued to exhibit vitritis and phlebitis on examination. She received two times dexamethasone intravitreal implants for local therapy in both eyes with a partial improvement of phlebitis and vitritis. She received later intravitreal fluocinolone injectable implants in both eyes. Despite receiving a long-acting steroid implant, chorioretinal inflammation persisted, showing active phlebitis on fluorescein angiography (FA), choroiditis on ICG angiography (ICGA), and choroidal thickening on optical coherence tomography (OCT) in both eyes. Twenty-one months later, the patient was placed on the selective JAK1 inhibitor, upadacitinib 45 mg daily, per an agreement between her uveitis specialist and rheumatologist. A progressive improvement was noted in her clinical findings over multiple ocular exams, including decreased vascular leakage/staining on FA and early and late hypofluorescent spots on ICGA, representing improved retinal phlebitis and choroiditis [Figure 1]. Her vision also improved from 20/40 to 20/30 in the right eye and 20/30 to 20/25 in the left eye, secondary to resolved vitreous haze after 11 months. The patient did not experience a significant systemic side effect secondary to upadacitinib.

### Case 2

A 59-year-old female with HLA-B27-positive Crohn's disease was referred for bilateral chronic anterior and intermediate uveitis and persistent bilateral cystoid macular edema (CME). She was previously trialed on systemic immunotherapies, including methotrexate, etanercept, mycophenolate mofetil, and rituximab. She had also received local steroid injections and implants. She underwent surgical procedures to insert fluocinolone acetonide intravitreal implant in both eyes about 15 years ago, which was repeated in the left eye three years later. Her course was complicated by secondary glaucoma requiring trabeculectomy procedures in both eyes. At our initial evaluation, the patient was on four times daily difluprednate 0.05% drop in both eyes but not on systemic therapy. The best visual acuity was 20/200 and 20/50 in the right and left eyes, respectively. Intraocular pressure (IOP) was 5 mm Hg (right eye) and 3 mm Hg (left eye). The exam revealed moderate anterior chamber and vitreous inflammation, right optic nerve pallor, and bilateral epiretinal membranes with CME. Images from her prior retinal specialist revealed chronic peripheral retinal pigment mottling and optic nerve edema.

**Figure 2:**
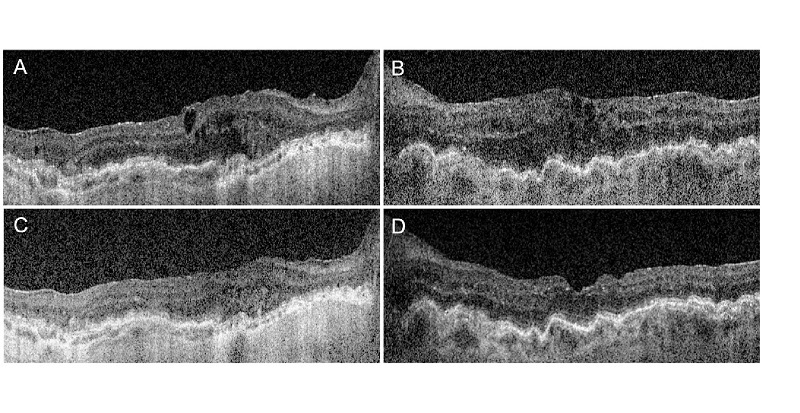
Enhanced depth imaging optical coherence tomography of the macula of the right and left eyes before (A, B, respectively) and right and left eyes after (C, D, respectively) initiation of upadacitinib, demonstrating improvement in CME bilaterally.

She was referred back to rheumatology to reinitiate systemic immunotherapy. The patient's rheumatologist initiated upadacitinib to cover her systemic conditions, including Crohn's disease and arthritis. Six months later, while she was only taking difluprednate 0.05% drop once a day in both eyes, a significant improvement in the anterior chamber and vitreous inflammation was detected on the slit lamp exam. The best corrected visual acuity was 20/70 in the right eye and 20/30 in the left eye, and IOPs were 8 mm Hg (right eye) and 5 mm Hg (left eye). The OCT showed improved CME from pretreatment visits but with persistent hypotony maculopathy [Figure 2].

##  DISCUSSION

Advances in molecular and translational biology have led to the detection of pathways and molecular targets that play a role in regulating immune responses and, secondarily, the development of many new drugs for managing autoimmune diseases. One is the JAK inhibitors family, which potentially reduces inflammatory responses by inhibiting the JAK/STAT system and its consequent pro-inflammatory effects. JAK inhibitors are generally classified into two generations. The first-generation JAK inhibitors were broadly active, inhibiting three or up to all four JAK members, such as tofacitinib and baricitinib, which have been found helpful in treating several inflammatory ocular diseases such as JIA-associated uveitis and scleritis in case reports.^[9,10,11,12,13]^ A phase 3 trial on baricitinib in pediatric anterior uveitis is ongoing.^[14]^

The second-generation JAK inhibitors exert more selective effects and are associated with fewer adverse events by inhibiting fewer JAK members. Upadacitinib is a second-generation JAK inhibitor with greater selectivity for JAK1 than for JAK2, JAK3, or TYK2. Upadacitinib was first approved for the treatment of moderate to severe RA in 2019 and later received approval for the treatment of atopic dermatitis, psoriatic arthritis, Crohn's disease, and ulcerative colitis.^[3,4,5,6,7,8][15][16]^ There have been a few case reports describing its successful use in recalcitrant anterior uveitis, intermediate uveitis, scleritis, a case of tofacitinib-refractory uveitis, and two cases of Behcet's panuveitis.^[17,18,19,20]^ The therapeutic effects of upadacitinib are thought to arise from its selective targeting of JAK1, which plays a pivotal role in the signal transduction of several proinflammatory cytokines, IL-6, IL-23, and interferon-gamma.^[21,22]^ Additionally, upadacitinib was found to suppress pathogenic CD4 T cell proliferation.^[23]^

The reported safety profile of upadacitinb, including any adverse event, an adverse event leading to discontinuation, major adverse cardiovascular event, venous thromboembolism (VTE), and infection, was comparable to other treatment options such as TNF- α blockers and IL antagonists in clinical trials for RA, ankylosing spondylitis, psoriatic arthritis, ulcerative colitis,and Crohn's disease.^[24]^ In the only study in RA that reported malignancy as an outcome at week 12, there was no statistically significant difference in the rate between upadacitinib and placebo.^[25]^ Upadacitinib-associated immunosuppression places patients at increased risk of infection, including tuberculosis, invasive fungal infection, and various viral infections, including reactivation of latent herpes zoster. However, the actual serious infection rates with the drug are relatively low ( ∼ 3.23 per 100 patient-years).^[26]^ Although an increased risk of herpes zoster reactivation has been proposed in less-JAK1-selective JAK inhibitors (e.g., tofacitinib) compared to tumor necrosis alpha inhibitors (e.g., adalimumab), more recent meta-analyses suggest that the more selective JAK1 inhibitor upadacitinib does not exhibit similar increased risk.^[27]^ Similarly, all JAK inhibitors carry black box warnings from the US Food and Drug Administration owing to an increased risk of all-cause mortality, major adverse cardiovascular events (MACE), and VTE. It should be noted, however, that these increased risks have only been directly observed with tofacitinib in the setting of RA. Indeed, trial data and long-term follow-up studies of baricitinib and upadacitinib have not shown an increased risk of either MACE or VTE;^[28]^ thus, clinicians should be cognizant of the most recent published evidence when discussing risks with patients and prescribing these drugs.

Laboratory monitoring is indicated for patients undergoing upadacitinib therapy. As this drug can rarely induce bone marrow suppression, baseline and routine complete blood counts are indicated. Furthermore, upadacitinib can increase serum lipid levels, thus increasing cardiovascular risk; therefore, baseline fasting serum lipid levels should be measured and repeated roughly every three months. Similarly, liver function abnormalities have been reported, and liver function tests should be measured at baseline and periodically after starting upadacitinib. Finally, given the risk of infection with upadacitinib, viral hepatitis screening and tuberculosis testing should be performed at baseline, and evaluation of signs/symptoms of infection should be done routinely. Skin examinations are recommended in patients at increased risk of skin cancer. There are no consensus recommendations for the precise frequency of laboratory monitoring of patients on upadacitinib; in our practice, we monitor patients at each clinic visit (roughly every three to four months).

Here, we reported two patients, an adult whose uveitis was associated with a systemic disease responsive to upadacitinib and an adult in whom upadacitinib successfully induced remission of BCR, an isolated ocular condition. To our knowledge, our BCR patient is the first case of BCR reported with a successful response to upadacitinib. Our understanding of the pathophysiology of BCR is limited to several unapproved signaling pathways; however, the presence of CD4+ and CD8+ T in the vitreous samples of BCR patients and the detection of T cells as the dominant cells in tissue samples of birdshot lesions might explain the response to upadacitinb which suppress the proliferation of some subgroups of pathogenic T cells.^[29]^

In summary, our knowledge of the pathogenesis of uveitic diseases is lacking, and we need to know if this new group of biologics can enhance the therapeutic approaches for treating these vision-threatening conditions. Therefore, further translational research and prospective controlled clinical studies are required to confirm the therapeutic benefits of JAKininbs in uveitis, whether associated with systemic autoimmune diseases or isolated ocular inflammations such as BCR.

##  Financial Support and Sponsorship

None.

##  Conflicts of Interest

None.
